# WeatherMAR: Complementary Masking of Paired Tokens for Adverse-Weather Image Restoration

**DOI:** 10.3390/jimaging12040154

**Published:** 2026-04-02

**Authors:** Junyuan Ma, Qunbo Lv, Zheng Tan

**Affiliations:** 1 Aerospace Information Research Institute, Chinese Academy of Sciences, No. 9 Dengzhuang South Road, Haidian District, Beijing 100094, China; 2 School of Optoelectronics, University of Chinese Academy of Sciences, No. 19(A) Yuquan Road, Shijingshan District, Beijing 100049, China; 3 Department of Key Laboratory of Computational Optical Imaging Technology, Chinese Academy of Sciences, No. 9 Dengzhuang South Road, Haidian District, Beijing 100094, China

**Keywords:** adverse-weather restoration, masked autoregressive modeling, continuous visual tokens, conditional diffusion

## Abstract

Image restoration under adverse weather conditions has attracted increasing attention because of its importance for both human perception and downstream vision applications. Existing methods, however, are often designed for a single degradation type. We present **WeatherMAR**, a multi-weather restoration framework that formulates adverse-weather restoration as a paired-domain completion problem in a shared continuous token space. Specifically, WeatherMAR concatenates degraded and clean token sequences into a joint paired-domain sequence and performs restoration through masked autoregressive modeling, in which self-attention enables direct cross-domain interaction. To strengthen conditional learning while avoiding trivial paired correspondences, we introduce complementary bidirectional masking together with an optional reverse objective used only during training to encourage degradation-aware representations. WeatherMAR further employs a conditional diffusion objective for continuous token prediction and adopts a progress-to-step schedule to improve inference efficiency. Extensive experiments on standard multi-weather benchmarks, including Snow100K, Outdoor-Rain, and RainDrop, show that WeatherMAR achieves the best PSNR/SSIM on Snow100K-S (38.14/0.9684), the best SSIM on Outdoor-Rain (0.9396), and the best PSNR on Snow100K-L (32.58) and RainDrop (33.12). These results demonstrate that paired-domain token completion provides an effective solution for adverse-weather restoration.

## 1. Introduction

Image restoration under adverse weather conditions, including rain streaks, snow accumulation, and adherent raindrops, has long been a central problem in computer vision because of its scientific importance and practical relevance. In real-world settings, weather-induced degradations can obscure scene content, reduce contrast, and distort local structures, thereby impairing both human visual perception and downstream vision systems. Consequently, adverse-weather restoration remains a challenging inverse problem. The degradations are often spatially varying and entangled with scene textures, and can lead to the loss of fine details. Effective restoration must therefore remove diverse artifacts while faithfully recovering the underlying scene content. Over the years, substantial progress has been achieved in task-specific image restoration, including single-image deraining [1,2,3], dehazing [4,5,6,7], desnowing [8,9], and raindrop removal [10,11]. Although these methods often achieve strong in-domain performance, they are typically designed for a single degradation type and therefore struggle to handle heterogeneous weather degradations or large variations in weather effects across scenes.

To improve practicality, recent work has explored multi-weather restoration with shared model designs that handle diverse degradations within a common framework. Representative approaches include transformer-based encoder–decoder architectures [12,13], factorized models that separate weather-general and weather-specific components [14], and diffusion-based restoration frameworks [15,16]. These methods are typically evaluated under the standard multi-weather benchmark setting widely adopted by the community, which provides a common basis for fair comparison across representative adverse-weather restoration tasks. Despite this progress, multi-weather models still face a fundamental challenge: restoration must remain strongly conditioned on corrupted observations while avoiding trivial solutions under paired supervision. Superficial correspondences between paired inputs can reduce the need to learn degradation-aware representations, and a shared framework must still preserve fine structural details while removing diverse artifacts across weather types and severity levels.

To address these challenges, we propose WeatherMAR, a framework that formulates adverse-weather restoration as paired-domain completion in a shared continuous token space. Rather than predicting pixels directly, we encode degraded and clean images using a shared, frozen VAE tokenizer [17] and perform restoration on the resulting continuous tokens in the latent space. Building on masked autoregressive modeling with continuous tokens [18], we concatenate degraded and clean token sequences into a joint paired-domain sequence and use self-attention to enable direct cross-domain interaction. During inference, the model observes only degraded tokens, initializes all clean-token positions with [MASK], and progressively predicts the missing clean tokens conditioned on the degraded evidence, thereby providing a unified restoration interface across different weather degradations.

A key challenge in joint modeling is avoiding trivial solutions when paired information is fully visible. WeatherMAR addresses this issue through complementary bidirectional masking: at each spatial location, exactly one token in the degraded–clean pair is masked, while its counterpart remains visible. This strict location-wise constraint preserves strong conditional evidence at every position and prevents weakly conditioned predictions. It also enables an optional reverse objective during training, in which masked degraded tokens are predicted from visible clean tokens and weighted by λ to regularize degradation-aware representations. To model the conditional distribution of continuous tokens, WeatherMAR adopts a conditional token diffusion objective [18,19], with transformer features serving as conditioning signals for denoising-based token generation. Because diffusion-enhanced masked autoregressive inference can be computationally demanding under a fixed reverse-step budget, we further introduce a progress-to-step schedule that allocates more reverse diffusion steps to early, high-uncertainty iterations and fewer steps to later iterations, thereby improving efficiency while maintaining restoration quality.

We evaluate WeatherMAR on three standard adverse-weather benchmarks that cover diverse degradations: Snow100K [8], Outdoor-Rain [20], and RainDrop [10]. WeatherMAR achieves strong performance across these benchmarks, obtaining the best PSNR/SSIM on Snow100K-S (38.14/0.9684), the best SSIM on Outdoor-Rain (0.9396), and the best PSNR on Snow100K-L (32.58) and RainDrop (33.12) under the standard evaluation setting. We further compare WeatherMAR with strong multi-weather baselines, including TransWeather [13], WGWSNet [14], WeatherDiff [15], Histoformer [21] and CyclicPrompt [22].

Our contributions are summarized as follows:

- We propose WeatherMAR, a framework that formulates adverse-weather restoration as paired-domain completion in a shared continuous token space. By concatenating degraded and clean tokens into a joint sequence, the model enables direct cross-domain interaction through self-attention within a unified token-processing pipeline, without requiring additional fusion branches.
- We introduce complementary bidirectional masking, which enforces a strict location-wise constraint such that exactly one token in each degraded–clean pair is masked. This design preserves strong conditional evidence at every position, mitigates trivial correlations under paired supervision, and supports an optional reverse objective used only during training to encourage degradation-aware representations.
- We develop a progress-to-step guided sampling strategy to accelerate diffusion-enhanced masked autoregressive inference. This schedule allocates more denoising steps to early, high-uncertainty iterations and fewer steps to later iterations, thereby reducing redundant computation while maintaining restoration quality.

## 2. Related Work

### 2.1. Image Restoration in Adverse Weather Conditions

Over the past decade, adverse-weather restoration has advanced rapidly, driven by learning-based methods developed to model and remove weather-induced degradations [23,24,25,26,27,28,29,30,31] and to improve the perceptual quality of images and videos [32,33,34,35,36,37,38]. Most existing approaches target a single degradation type and are optimized for task-specific settings, such as single-image deraining [2,39,40,41], dehazing [4,5,6,7], desnowing [8,9,42,43], and raindrop removal [10,11,44,45]. More recently, multi-weather models have been introduced to handle multiple degradations within a single framework, with the goal of improving robustness and practicality under diverse real-world conditions [13,15,46]. Despite this progress, developing a single model that generalizes reliably across heterogeneous weather degradations while preserving fine structures and natural appearance remains challenging.

**Removing Raindrops.** Single-image raindrop removal has been studied extensively, encompassing both classical pipelines based on hand-crafted priors and modern learning-based approaches. Early studies explored the use of temporal redundancy for video-based raindrop removal [44]. For still images, early learning-based methods investigated supervised CNN-based restoration using paired raindrop-degraded and clean images, although the reconstructed results were often over-smoothed. Subsequent work introduced dedicated datasets and attention-based frameworks to better localize and suppress raindrop regions while recovering background content [10]. Building on this line, later methods further improved localization by incorporating edge-aware cues or explicit raindrop representations, thereby enhancing boundary handling and detail recovery around droplet contours [11].
**Image Desnowing.** Early deep-learning approaches to image desnowing typically treated snow as a learnable corruption and trained direct mappings from snowy inputs to clean targets. DesnowNet [8] is a representative CNN-based method that established paired-data learning for snow removal. Later studies showed that architectures originally developed for related restoration tasks can be effectively adapted to desnowing. For example, SPANet and RESCAN [3,47] achieve strong performance on synthetic snow benchmarks. To better account for diverse snow appearances, Chen et al. [48] proposed JSTASR, which explicitly models different snow characteristics within a unified framework. Zhang et al. [9] introduced DDMSNet, a dense multi-scale network that leverages auxiliary cues to improve robustness under heavy snow and has demonstrated strong performance in prior studies.
**Image Deraining & Dehazing.** Traditional single-image deraining methods relied on hand-crafted priors and decomposition, whereas modern approaches use deep networks to suppress rain streaks while preserving fine details [1,49]. Recurrent or iterative designs improve robustness by progressively estimating rain layers and refining the clean image over multiple steps, which is particularly helpful when rain streaks vary in scale and density [2]. In real heavy-rain scenarios, rain streaks often co-exist with haze-like veiling, making joint deraining–dehazing more effective than treating the two degradations independently. Representative methods explicitly model the coupled “streak + veil” degradation and recover visibility and contrast together with rain streak suppression [20]. To mitigate the synthetic-to-real gap, several studies have explored transfer and adaptation strategies that better align training data with real rainy images [41]. DerainCycleGAN [50] further investigates rain-attentive cycle-consistent translation for unsupervised single-image deraining, helping to alleviate the synthetic-to-real gap. More recently, transformer-based restoration models have leveraged long-range context to improve structural and textural coherence, and have been adopted or extended for unified adverse-weather restoration [13,26].
**Multi-Weather Restoration.** Beyond task-specific restoration, recent studies have explored multi-weather restoration, in which a shared framework is designed to handle multiple weather-related degradations. Valanarasu et al. [13] introduced TransWeather, a transformer-based encoder–decoder that learns a unified restoration mapping across multiple atmospheric degradations. Zhu et al. [14] developed WGWSNet, which separates weather-general and weather-specific representations through a staged training procedure. More recently, multi-weather restoration has been studied from several additional perspectives, including knowledge distillation [51], diffusion-based probabilistic restoration [15], prior- or codebook-based modeling [16], prompt-based conditioning [22], and grid-structured feature interaction [52]. Related all-in-one restoration studies have also explored broader settings beyond the standard multi-weather benchmark protocol, including expert routing and degradation embedding [53], perception-guided coarse-to-fine restoration [54], and continual weather restoration with dynamic expert libraries [55]. Among the methods evaluated under the standard multi-weather benchmark setting, recent models such as CyclicPrompt [22] and GridFormer [52] serve as strong baselines for comparison. Despite these advances, multi-weather restoration still faces the challenge of reliably conditioning restoration on diverse weather-corrupted observations while preserving fine details. To address this challenge, we propose WeatherMAR, which performs paired-domain completion in a shared latent token space through joint-sequence self-attention and complementary masking, and further refines predictions with conditional token diffusion.

### 2.2. Autoregressive Models with Continuous Tokens

Autoregressive (AR) generation has achieved remarkable success in sequence modeling [56,57,58,59], but extending it to images typically requires either pixel-space factorization or latent tokenization. Early AR vision models generate images by predicting pixels sequentially (e.g., iGPT [60]). A dominant line of work discretizes images into codebook tokens through learned vector quantization [61], thereby enabling transformer-based generation over discrete latent sequences [62,63,64]. However, discretization relies on a finite codebook and may introduce information loss or approximation errors, motivating autoregressive generation over continuous-valued token sequences. Recent efforts have explored real-valued or hybrid token representations to improve fidelity and efficiency, including continuous token synthesis, coarse-to-fine decompositions [65,66], and unified token spaces for multimodal generation [67,68,69].

Beyond standalone image generation, diffusion has also been adopted as a learning objective and as a conditional decoder in other settings. In visual self-supervised learning, diffusion-based decoders have been used to reconstruct masked content and improve representation quality. For example, prior work has replaced the standard MAE reconstruction loss [70] with a denoising diffusion decoder [71] or trained AR-style backbones with diffusion patch decoders [72]. Although these studies primarily target representation learning rather than diverse image synthesis, they highlight the ability of diffusion models to capture complex conditional distributions in continuous spaces. Diffusion has also been explored in decision-making settings, where actions are modeled as conditional denoising processes given observations [73]. Motivated by these advances, recent masked and iterative generation frameworks have integrated diffusion heads with autoregressive-style token prediction in continuous latent spaces [18]. However, this combination often incurs substantial sampling cost, thereby motivating the development of more efficient scheduling strategies for iterative generation.

## 3. Methodology

### 3.1. Overall Framework

Image restoration under adverse weather conditions aims to recover a clean image from observations degraded by weather effects, such as rain and snow. Given a degraded input $y\in R^{H\times W\times 3}$, the goal is to reconstruct its corresponding clean image $x\in R^{H\times W\times 3}$. We consider a paired supervised setting with the training data defined as:

(1) $$D={\left\{\left(y^{\left(n\right)},x^{\left(n\right)}\right)\right\}}_{n=1}^{N_{\mathrm{pair}}},$$

where $N_{\mathrm{pair}}$ denotes the number of paired degraded–clean training samples and *n* indexes an individual training pair. Based on these paired data, we learn a conditional generative model that predicts $\hat{x}$ from *y*, removing weather degradations while preserving fine details and natural appearance. During inference, the model takes only the degraded image *y* as input, initializes the clean-token positions with mask tokens, and progressively predicts the missing clean tokens to produce the restored image $\hat{x}$.

**Figure 1:**
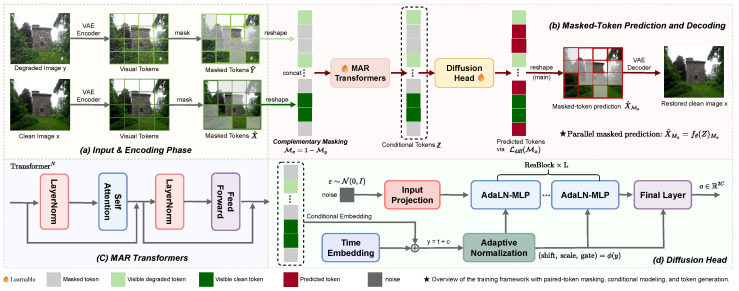
Overview of the WeatherMAR training framework. (**a**) Paired degraded and clean images are encoded into continuous tokens by a shared VAE tokenizer and then masked in a complementary manner. (**b**) The masked tokens are concatenated into a joint sequence for paired-domain modeling. (**c**) MAR transformers compute conditional representations for the masked positions. (**d**) A conditional diffusion head predicts the masked tokens, which are then decoded into the restored clean image.

WeatherMAR introduces a new restoration paradigm for spatially aligned paired adverse-weather benchmarks by formulating restoration as paired-domain masked token completion in a shared latent space. Complementary masking explicitly couples degraded and clean tokens at each spatial location. As illustrated in Figure 1, WeatherMAR performs restoration in a continuous latent token space. A shared tokenizer E(·) [17] maps *y* and *x* into latent token grids $Y,X\in R^{h\times w\times d}$, where h×w denotes the token-grid resolution and *d* is the token dimension. We flatten each grid into a sequence of length N=hw and concatenate the degraded and clean sequences for joint modeling, yielding a joint sequence of length 2N. This formulation enables degraded and clean representations to be modeled jointly within a unified token space. To strengthen conditional learning while discouraging trivial solutions, WeatherMAR applies complementary token masking. At each spatial location, exactly one token in each degraded–clean pair is masked, while the other remains visible. This complementary masking mechanism is central to the restoration design, as it preserves local cross-domain evidence while preventing shortcut learning from fully visible token pairs. The masked joint sequence is processed by a masked iterative transformer $f_{\theta }$ (MAR-style) [18] to aggregate global context and produce conditioning representations for the masked positions. A diffusion-based denoising head then models the conditional distribution of the masked tokens and refines their estimates. The refined tokens are reshaped into $R^{h\times w\times d}$ and decoded to obtain the restored image $\hat{x}$.

WeatherMAR is trained with paired inputs and complementary masking, providing coupled supervision over masked subsets in both domains. During inference, the model observes only *y*, initializes the clean-token positions with mask tokens, and progressively predicts the missing clean tokens conditioned on the visible degraded tokens. For efficiency, we adopt a progress-guided step schedule to allocate the diffusion sampling budget across inference iterations, using more denoising steps in earlier iterations and fewer in later ones.

### 3.2. Paired-Domain Joint Sequence Modeling

WeatherMAR formulates adverse-weather restoration as paired-domain completion over a unified sequence in a continuous latent token space, implemented with masked autoregressive modeling [18]. Unlike standard restoration methods that operate in pixel space or introduce conditions through separate branches, this formulation allows degraded and clean tokens to interact directly within a shared representation space. Given a paired sample (y,x), a shared tokenizer E(·) produces aligned token grids $Y,X\in R^{h\times w\times d}$, which are then flattened and concatenated into a joint sequence for cross-domain interaction.

We flatten each grid into a sequence of length N=hw, yielding two token sequences with identical spatial ordering:

(2) $$Y={\left\{y_{i}\right\}}_{i=1}^{N}\in R^{N\times d},\;X={\left\{x_{i}\right\}}_{i=1}^{N}\in R^{N\times d},$$

where *d* denotes the latent token dimension, each token index *i* corresponds to a specific spatial location, and the shared ordering implies that $y_{i}$ and $x_{i}$ originate from aligned positions in the degraded and clean images, respectively.

We concatenate the degraded and clean sequences to form a joint sequence:

(3) $$Z=\left[Y;X\right]\in R^{2N\times d},$$

where the first *N* tokens correspond to the degraded domain, and the remaining *N* tokens correspond to the clean domain. The key idea is to use self-attention over the unified sequence as the cross-domain fusion mechanism, allowing tokens from one domain to directly attend to those from the other while preserving location-wise correspondence.

This formulation enables joint masked-token modeling by predicting masked tokens in *Z* conditioned on the visible subset. In particular, completing masked clean tokens conditioned on visible degraded tokens implements conditional restoration and provides a practical way to learn the conditional distribution $p_{\theta }\left(X|Y\right)$. Compared with designs that inject conditional information through separate branches, the joint sequence integrates global context and cross-domain evidence within a single representation space, which helps disambiguate structures corrupted by adverse weather.

Let $\tilde{Z}$ denote the masked joint sequence constructed using the masking strategy described in Section 3.3. A masked iterative transformer $f_{\theta }$ takes $\tilde{Z}$ as input and outputs contextual representations for all tokens:

(4) $$H=f_{\theta }\left(\tilde{Z}\right)\in R^{2N\times d},$$

where the resulting representations aggregate global context across both domains and serve as conditioning signals for masked-token prediction. Each row of *H* corresponds to a token position in the joint sequence and encodes contextual information from both the degraded and clean domains. However, naive joint modeling may admit trivial solutions when paired information is always fully visible. We therefore introduce complementary bidirectional masking to enforce strong conditional completion and regularize cross-domain reasoning.

### 3.3. Complementary Bidirectional Masking Strategy

#### 3.3.1. Complementary Mask Construction

**Figure 2:**
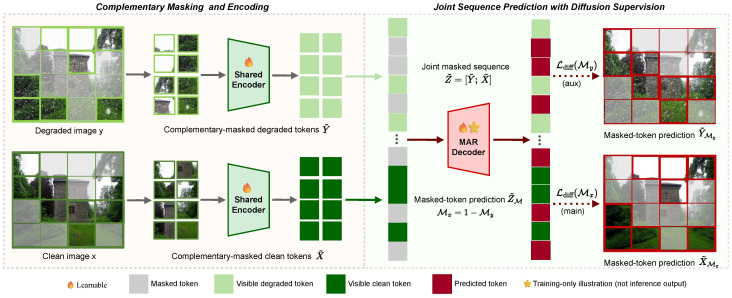
Complementary bidirectional masking for WeatherMAR training, corresponding to the training components shown in Figure 1a,b. Paired degraded and clean tokens are masked in a complementary manner: at each spatial location, one domain token is masked while the other remains visible. The resulting tokens are concatenated into a masked joint sequence for paired-domain modeling, yielding two coupled training signals: restoration from degraded to clean tokens and reverse degradation modeling from clean to degraded tokens.

As shown in Figure 2, a key design of WeatherMAR is a complementary masking mechanism that preserves strong conditional evidence while discouraging trivial solutions. Under adverse weather conditions, corrupted observations entangle scene content with degradation patterns. If masked prediction lacks sufficient local conditioning, learning becomes ambiguous and may drift toward unconditional token generation. Conversely, if both domains are always visible, the model may overfit to trivial correlations, thereby reducing the incentive for cross-domain reasoning. Complementary masking addresses both issues by enforcing a strict local constraint: at each spatial location, one domain provides observable evidence, whereas the other must be inferred.

Formally, given the token sequences *Y* and *X* in Equation (2), we sample a binary mask for the clean-domain tokens:

(5) $$M_{x}\left[i\right]\overset{i.i.d.}{∼}\mathrm{Bernoulli}\left(r\right),\;i=1,\ldots ,N,\;M_{x}\in {\left\{0,1\right\}}^{N},$$

where r∈(0,1) denotes the masking probability for clean-domain tokens and N=hw is the sequence length. $M_{x}\left[i\right]=1$ indicates that the clean token $x_{i}$ is masked and must be predicted, whereas $M_{x}\left[i\right]=0$ indicates that $x_{i}$ remains visible as context. We then define the degraded-domain mask as the complement of the clean-domain mask:

(6) $$M_{y}=1_{N}-M_{x},$$

where $1_{N}$ denotes the all-ones vector of length *N*. This complementary construction guarantees that the token pair $\left(y_{i},x_{i}\right)$ at location *i* is never masked simultaneously. Equivalently, $M_{x}\left[i\right]+M_{y}\left[i\right]=1$ holds for all *i*, ensuring that one domain always provides local evidence.

Let $\left[\mathrm{MASK}\right]\in R^{d}$ denote a shared learnable mask embedding. The complementarily masked token sequences are constructed as follows:

(7) $$\tilde{X}=\left(1_{N}-M_{x}\right)⊙X+M_{x}⊙\left[\mathrm{MASK}\right],\;\tilde{Y}=\left(1_{N}-M_{y}\right)⊙Y+M_{y}⊙\left[\mathrm{MASK}\right],$$

where ⊙ denotes element-wise multiplication with broadcasting along the token dimension. Masked positions are replaced by the learnable mask embedding, whereas visible positions retain the original latent tokens. Following Equation (3), we form the masked joint sequence:

(8) $$\tilde{Z}=\left[\tilde{Y};\tilde{X}\right]\in R^{2N\times d},$$

which is then fed to the transformer backbone for contextual inference. This complementary constraint ensures that the backbone always observes a visible counterpart token at each spatial location, thereby stabilizing conditional completion in the paired sequence space.

#### 3.3.2. Bidirectional Completion Targets

Complementary masking induces two coupled completion targets within a single forward pass. We define masked index sets for the clean and degraded domains as follows:

(9) $$M_{x}=\left\{i|M_{x}\left[i\right]=1\right\},\;M_{y}=\left\{i|M_{y}\left[i\right]=1\right\}.$$

$M_{x}$ and $M_{y}$ therefore denote the spatial locations where clean and degraded tokens are masked, respectively. By construction, these sets form a disjoint partition of {1,…,N}:

(10) $$M_{x}\cap M_{y}=⌀,\;M_{x}\cup M_{y}=\left\{1,\ldots ,N\right\},$$

which means each spatial location contributes supervision to exactly one domain, while the other domain provides paired evidence. Note that $M_{x}$ and $M_{y}$ index spatial locations rather than individual tokens in the joint sequence. At each location, only one domain token is masked, so the number of masked tokens in the joint sequence is *N* rather than 2N.

The main restoration objective predicts clean tokens at positions $M_{x}$ conditioned on visible degraded tokens and the global context aggregated from $\tilde{Z}$. Because $M_{x}\left[i\right]=1$ implies $M_{y}\left[i\right]=0$, the degraded token $y_{i}$ at the same location remains visible and provides a strong local cue for inferring the missing clean content, thereby implementing the restoration mapping y→x at the token level. Symmetrically, we introduce an auxiliary reverse objective that predicts degraded tokens at $M_{y}$ conditioned on visible clean tokens.

This x→y task encourages the backbone to explicitly encode weather degradation factors (e.g., rain and snow patterns) in the shared token space, rather than absorbing them implicitly as residual noise, thereby promoting more robust modeling of diverse degradation patterns. When optimized jointly with the main y→x objective under complementary masking, the reverse direction provides coupled supervision at every spatial location and regularizes cross-domain correspondence learning without introducing any additional components or computation at test time.

#### 3.3.3. Training and Inference Separation

The complementary masking strategy provides supervision over two disjoint token subsets within a single masked joint sequence, without introducing additional architectural branches. This design enables bidirectional supervision during training while preserving unidirectional restoration during inference. During training, WeatherMAR jointly optimizes masked-token prediction over $M_{x}$ and $M_{y}$, with the overall objective defined as a weighted sum over the two subsets:

(11) $$L_{\mathrm{total}}=L_{\mathrm{mask}}\left(M_{x}\right)+\lambda \;L_{\mathrm{mask}}\left(M_{y}\right),$$

where $L_{\mathrm{mask}}\left(\cdot \right)$ denotes a masked-token generative loss evaluated on the specified index subset, and λ controls the trade-off between the auxiliary reverse-direction objective and the main restoration objective. We set λ=1 in all experiments. We instantiate $L_{\mathrm{mask}}\left(\cdot \right)$ as a diffusion-based conditional denoising objective, as described in Section 3.4. Intuitively, the first term corresponds to recovering masked clean tokens from visible degraded evidence, while the second encourages the model to encode degradation factors by predicting masked degraded tokens from visible clean tokens.

During inference, WeatherMAR observes only *y*, initializes all clean-token positions with [MASK], and progressively generates clean tokens conditioned on the degraded tokens. The reverse x→y objective is used only during training as an auxiliary loss on $M_{y}$ and introduces no additional components or computation at inference time.

### 3.4. Token Diffusion Objective with Conditional Denoising

WeatherMAR predicts continuous-valued visual tokens [18], for which masked-token generation is more naturally formulated as conditional distribution modeling rather than deterministic regression. As shown in MAR [18], diffusion-based learning effectively models per-token conditional distributions in continuous token space, thereby making sequence modeling compatible with continuous-valued visual tokens. This formulation is particularly important for adverse-weather restoration, where corrupted observations may correspond to multiple plausible clean latent reconstructions rather than a single deterministic target. Therefore, we adopt a conditional denoising diffusion objective [19] for masked-token prediction, with transformer outputs serving as informative conditioning signals for the denoising process.

**Conditional token distribution.** Let $\tilde{Z}\in R^{2N\times d}$ denote the masked joint sequence in Equation (8), and let $H=f_{\theta }\left(\tilde{Z}\right)\in R^{2N\times d}$ denote the contextual representation in Equation (4). Recall that Z=[Y;X] places degraded tokens in the first *N* positions and clean tokens in the last *N* positions. For a spatial index i∈{1,…,N}, the degraded-domain token corresponds to the joint index $\pi_{y}\left(i\right)=i$, and the clean-domain token corresponds to $\pi_{x}\left(i\right)=N+i$. For each masked position, the transformer feature $h_{\pi (i)}\in R^{d}$ serves as a conditioning vector for token generation. We define the masked-token variable $u_{i}^{0}\in R^{d}$ as follows:
  (12) $$u_{i}^{0}=\left\{\begin{array}{cc} x_{i}, & i\in M_{x},\\ y_{i}, & i\in M_{y}, \end{array}\right.$$
  where $M_{x}$ and $M_{y}$ are defined in Equation (9). The corresponding conditioning feature is defined as follows:
  (13) $$c_{i}=\left\{\begin{array}{cc} h_{\pi_{x}\left(i\right)}, & i\in M_{x},\\ h_{\pi_{y}\left(i\right)}, & i\in M_{y}, \end{array}\right.$$
  This formulation provides a unified prediction interface across domains while remaining domain-aware through the joint index.
**Forward noising process.** For each masked token $u_{i}^{0}$, we uniformly sample a diffusion step t∈{1,…,T} and add Gaussian noise:
  (14) $$u_{i}^{t}=\sqrt{{\bar{\alpha }}_{t}}\;u_{i}^{0}+\sqrt{1-{\bar{\alpha }}_{t}}\;\epsilon_{i},\;\epsilon_{i}∼N\left(0,I\right),$$
  where ${\left\{{\bar{\alpha }}_{t}\right\}}_{t=1}^{T}$ denotes a predefined noise schedule and $u_{i}^{t}$ denotes the noisy token at step *t*. Specifically, ${\bar{\alpha }}_{t}=\prod_{s=1}^{t}\alpha_{s}$ is the cumulative noise coefficient induced by the predefined schedule ${\left\{\alpha_{s}\right\}}_{s=1}^{T}$. As *t* increases, $u_{i}^{t}$ becomes progressively less informative about $u_{i}^{0}$.
**Conditional denoising objective.** A lightweight denoising head $\epsilon_{\theta_{d}}\left(\cdot \right)$ takes $\left(u_{i}^{t},t,c_{i}\right)$ as input and predicts the added noise. We minimize a noise-prediction objective over a masked index set S:
  (15) $$L_{\mathrm{diff}}\left(S\right)=E_{t,\epsilon }\left[\frac{1}{\left|S\right|}\sum_{i\in S}{∥\epsilon_{i}-\epsilon_{\theta_{d}}\left(u_{i}^{t},t,c_{i}\right)∥}_{2}^{2}\right],$$
  where |S| is the cardinality of S. Minimizing Equation (15) trains a shared denoiser to model the conditional distribution of the masked tokens. Gradients are backpropagated through $c_{i}$ to jointly optimize the transformer parameters θ and the denoising-head parameters $\theta_{d}$.
**Main and auxiliary objectives.** Using Equation (15), we define two directional losses by evaluating the same diffusion objective on two disjoint index subsets:
  (16) $$L_{y\to x}≜L_{\mathrm{diff}}\left(M_{x}\right),\;L_{x\to y}≜L_{\mathrm{diff}}\left(M_{y}\right),$$
  These correspond to predicting masked clean tokens conditioned on visible degraded tokens and masked degraded tokens conditioned on visible clean tokens, respectively. Both terms share the same joint sequence and model parameters and differ only in the index subset used for loss evaluation. Substituting Equation (16) into Equation (11) instantiates $L_{\mathrm{mask}}\left(\cdot \right)$ as a diffusion-based masked-token objective.
**Sampling.** During inference, for each masked token, we run the reverse diffusion process conditioned on $c_{i}$, starting from Gaussian noise $u_{i}^{T}∼N\left(0,I\right)$ and producing ${\hat{u}}_{i}^{0}$ after $S_{k}$ reverse steps, where $S_{k}$ denotes the number of reverse diffusion steps allocated to the *k*-th inference iteration according to the progress-to-step schedule in Section 3.5. The sampled tokens are placed back into the clean-token positions and decoded into the image space. During restoration inference, we keep the degraded tokens from the input *y* fixed and generate only the missing clean tokens, consistent with the y→x direction.

### 3.5. Progress-to-Step Guided Sampling for Efficient Inference

**Figure 3:**
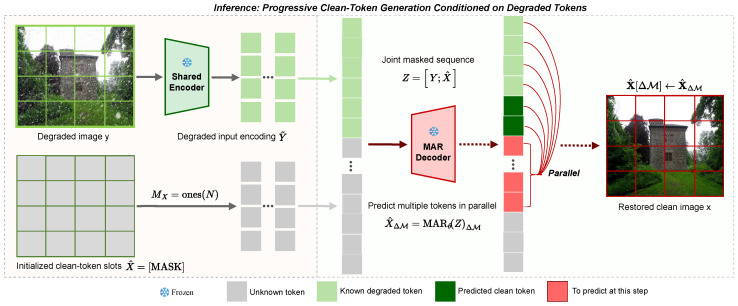
Inference procedure of WeatherMAR, illustrating the inference-time counterpart of the paired-token formulation shown in Figure 1a,b. Given a degraded image *y*, the model encodes it into degraded tokens *Y*, initializes the clean-token positions with [MASK], and progressively predicts the missing clean tokens conditioned on *Y*. At each iteration, a subset of clean tokens is generated in parallel by conditional diffusion and written back to update the clean-token positions.

WeatherMAR performs restoration through MAR iterative diffusion sampling, making inference efficiency an important practical consideration. Accordingly, we introduce a model-specific sampling strategy tailored to this inference process, rather than a generic acceleration scheme applicable to other restoration baselines. As illustrated in Figure 3, given a degraded input *y*, we compute degraded tokens $Y=E\left(y\right)\in R^{N\times d}$ using the shared tokenizer [17], and initialize the clean-token positions ${\hat{X}}^{\left(0\right)}\in R^{N\times d}$ by filling all positions with the learnable mask embedding [MASK]. At iteration *k*, we form the joint sequence:

(17) $${\hat{Z}}^{\left(k\right)}=\left[Y;{\hat{X}}^{\left(k\right)}\right]\in R^{2N\times d},$$

and obtain the contextual representations $H^{\left(k\right)}=f_{\theta }\left({\hat{Z}}^{\left(k\right)}\right)$.

Following MAR-style masked iterative completion, WeatherMAR progressively predicts missing clean tokens in parallel. Let $\Delta M^{\left(k\right)}\subseteq \left\{1,\ldots ,N\right\}$ denote the set of clean-token indices generated at iteration *k*. We select $\Delta M^{\left(k\right)}$ using the MAR-style cosine masking-ratio schedule with randomized order [18]. For each $i\in \Delta M^{\left(k\right)}$, we sample a token by running the reverse diffusion process conditioned on the corresponding feature in $H^{\left(k\right)}$ (Section 3.4) and write it back to update the clean-token positions:

(18) $${\hat{X}}^{\left(k+1\right)}\left[i\right]=\left\{\begin{array}{cc} {\hat{u}}_{i}^{\left(k\right)}, & i\in \Delta M^{\left(k\right)},\\ {\hat{X}}^{\left(k\right)}\left[i\right], & \mathrm{otherwise}, \end{array}\right.$$

where ${\hat{u}}_{i}^{\left(k\right)}\in R^{d}$ denotes the clean token generated at position *i* during iteration *k*. After *K* iterations, we decode the restored image as $\hat{x}=D\left({\hat{X}}^{\left(K\right)}\right)$.

A straightforward implementation allocates a fixed number of reverse diffusion steps to each iteration, which can be inefficient because the conditional context becomes progressively more informative as more clean tokens are predicted. We therefore propose a progress-to-step schedule that allocates the sampling budget across inference iterations. Specifically, we define the normalized inference progress at iteration *k* as:

(19) $$p_{k}=\frac{k}{K-1},\;k=0,1,\ldots ,K-1,\;\left(K>1\right),$$

and set the number of reverse diffusion steps using a monotonically decreasing schedule:

(20) $$S_{k}=\left\lceil S_{\min}+\left(1-p_{k}\right)\left(S_{\max}-S_{\min}\right)\right\rceil ,$$

where $S_{\max}=50$ and $S_{\min}=5$ denote the maximum and minimum numbers of reverse-diffusion steps per iteration, respectively. This design allocates more denoising steps to earlier iterations, when predictions rely on limited clean context and therefore exhibit higher uncertainty, and gradually reduces the step budget as the joint context becomes more informative and conditional completion becomes increasingly reliable.

As a result, WeatherMAR allocates more computation to earlier, more uncertain iterations and reduces redundant denoising in later ones, improving inference efficiency while preserving restoration quality. This schedule complements the paired-token restoration framework by making iterative conditional sampling more efficient during inference.

## 4. Experiments

### 4.1. Datasets and Evaluation Metrics

WeatherMAR is evaluated on the following community-standard paired adverse-weather benchmarks, for which established training and test protocols and prior baselines enable meaningful and fair comparison. We evaluate WeatherMAR on three adverse-weather restoration datasets covering diverse degradations, including synthetic snow with severity-controlled splits, heavy rain accompanied by haze, and raindrops attached to the camera lens. These datasets provide paired degraded and clean images for quantitative evaluation, and we additionally use a real-image subset to assess real-world generalization.

**Snow100K** [8] is a standard benchmark for image desnowing. It contains 50,000 training pairs and 50,000 test pairs. The synthetic test set is divided into three subsets, Snow100K-S/M/L, corresponding to light, medium, and heavy snow, with 16,611, 16,588, and 16,801 images, respectively. Snow100K also includes 1329 real snowy images (Snow100K-Real) without paired ground truth, which we use to assess real-world generalization.
**Outdoor-Rain** [20] targets joint deraining and dehazing under heavy-rain conditions. The training set contains 9000 paired images. For evaluation, we follow the standard protocol and report results on the Test1 split, which contains 750 image pairs.
**RainDrop** [10] focuses on raindrops adhered to the camera sensor or lens, which introduce localized occlusion-like artifacts. The dataset includes 861 training image pairs. For quantitative evaluation, we adopt the standard RainDrop-A test subset, which contains 58 image pairs and has been used in prior work for consistent comparison.
**Evaluation metrics.** We report peak signal-to-noise ratio (PSNR) [74] and structural similarity (SSIM) [75] on the paired test sets. Following common image-restoration practice, we compute PSNR and SSIM on the luminance channel *Y* in the YCbCr color space for fair comparison, in accordance with prior convention [10,76,77]. To evaluate real-world restoration quality in the absence of ground truth, we additionally employ two no-reference image quality metrics, NIQE [78] and IL-NIQE [79]. Lower NIQE and IL-NIQE values indicate better perceptual image quality.

### 4.2. Training Details

**Tokenizer and inputs.** We use a shared, frozen KL-regularized VAE tokenizer with a downsampling factor of 16 (KL-16) [17] for both degraded and clean images. All experiments and model variants in this work use the same frozen KL-16 tokenizer. Following standard MAR-style continuous latent modeling [18], we treat KL-16 as a fixed image-to-token interface rather than as a research variable or contribution of this paper. Consequently, the performance differences reported in this work primarily reflect the restoration design of WeatherMAR rather than differences in tokenizer design. For 256×256 inputs, the tokenizer outputs a continuous latent grid in $R^{h\times w\times d}$ with h×w=16×16 (N=hw=256), which is then flattened into token sequences. During training, we extract aligned 256×256 crops from each degraded–clean pair to preserve pixel-wise correspondence.
**Backbone and diffusion head.** We adopt a MAR-style masked iterative transformer with a joint-sequence length of 2N and learnable positional embeddings. We use the mar_large [18] configuration, with embedding dimension 1024, depth 16, 16 attention heads, and an MLP ratio of 4, together with attention dropout of 0.1 and projection dropout of 0.1. Masked positions are represented by a learnable mask token. Masked-token generation uses a conditional diffusion head implemented as an AdaLN-conditioned MLP with depth 12 and width 1536, conditioned on transformer features at the corresponding joint indices. We adopt a diffusion head that follows the standard MAR design for continuous-valued visual tokens [18]. This choice is well aligned with WeatherMAR, which performs masked-token prediction in a continuous latent space and therefore benefits from conditional distribution modeling rather than deterministic token regression.
**Optimization.** All experiments are implemented in PyTorch 2.8.0+cu128 [80] and trained on an NVIDIA RTX 4090 GPU. We use AdamW [81] with a learning rate of $1\times 10^{-4}$, weight decay of 0.02, and $\left(\beta_{1},\beta_{2}\right)=\left(0.9,0.95\right)$. We train for 400 epochs with a batch size of 16, enable mixed-precision training with bfloat16, apply gradient clipping with a threshold of 1.0, and maintain an exponential moving average (EMA) of the model parameters with a decay of 0.9999 for evaluation. For the main comparisons in Table 1, we follow the standard benchmark protocols for Snow100K, Outdoor-Rain, and RainDrop by training and evaluating WeatherMAR separately on each dataset under its corresponding setting. This ensures that comparisons are conducted under the same dataset-specific protocol as prior methods.
**Masking and inference defaults.** During training, we apply complementary bidirectional masking (Section 3.3) with a masking ratio of r=0.5 to sample $M_{x}$ and set $M_{y}=1-M_{x}$. This ensures each spatial location contributes supervision to exactly one domain and that the joint sequence contains *N* masked tokens in each forward pass. During inference, the model observes only *y*, keeps the degraded tokens fixed, initializes all clean-token positions with [MASK], and performs MAR parallel completion for K=64 iterations using a cosine unmasking schedule with randomized order [18]. Unless otherwise specified, we use the progress-to-step schedule (Equation (20)) with $S_{\max}=50$ and $S_{\min}=5$ in all reported results.

### 4.3. Multi-Weather Image Restoration Results

#### 4.3.1. Comparison Baselines and Protocol

We evaluate WeatherMAR on three standard adverse-weather restoration benchmarks that cover representative weather degradations: image desnowing (Snow100K-S/L) [8], joint deraining and dehazing (Outdoor-Rain) [20], and raindrop removal (RainDrop) [10]. These benchmarks constitute a commonly adopted evaluation protocol in prior adverse-weather restoration literature and provide a representative testbed for assessing restoration performance across multiple weather conditions. For fair comparison, the results in Table 1 are obtained by training and evaluating separate models on each benchmark following the standard dataset-specific protocol, unless otherwise stated. Following common practice, we include two categories of baselines. *(i) Task-specific methods* [3,8,9,10,11,20,47,48,76,77,82,83,84,85,86,87,88] are trained for a single degradation type and evaluated on the corresponding benchmark, covering both convolutional and transformer-based restoration models. These approaches often excel in their target degradation, but they do not directly evaluate unified multi-weather restoration under a single model formulation. *(ii) Unified multi-weather models* [12,13,14,15,16,21,22,51,52,89] aim to restore multiple weather degradations with a single network and therefore provide the most relevant references for WeatherMAR. In particular, the unified part of Table 1 includes representative baselines ranging from earlier unified restoration models, such as TransWeather [13], WGWSNet [14], WeatherDiff [15], AWRCP [16], and Histoformer [21], to more recent methods, including GridFormer [52] and CyclicPrompt [22]. Unless otherwise stated, we follow the standard evaluation protocol of each dataset and report PSNR and SSIM on the paired test sets for fair comparison.

#### 4.3.2. Quantitative Comparison

As summarized in Table 1, WeatherMAR delivers strong performance among multi-weather models across all three tasks, achieving the best results on Snow100K-S, the highest SSIM on Outdoor-Rain, and the best PSNR on RainDrop and Snow100K-L.

On **Snow100K-S**, WeatherMAR achieves 38.14 dB/0.9684, surpassing the strongest unified baseline, T^3^-DiffWeather (37.55/0.9641), by 0.59 dB and improving upon the best baseline SSIM (0.9656, achieved by Histoformer) by 0.0028. On the more challenging **Snow100K-L** split, WeatherMAR achieves 32.58 dB/0.9274, improving upon the best unified baseline PSNR (32.16 dB, achieved by Histoformer and CyclicPrompt) by 0.42 dB. These gains on both light and heavy snow suggest that WeatherMAR remains effective across snow severities while preserving fine structures.

On **Outdoor-Rain**, WeatherMAR achieves 31.91 dB/0.9396. Although CyclicPrompt attains the highest PSNR (32.81 dB), ours achieves the highest SSIM, improving upon the previous best SSIM of 0.9389 reported by Histoformer. This result suggests improved structural fidelity and perceptual consistency under heavy rain and haze.

On **RainDrop**, WeatherMAR achieves the best PSNR (33.12 dB) together with a highly competitive SSIM (0.9452). It slightly exceeds Histoformer in PSNR (33.06 dB) while remaining marginally below the best unified SSIM reported by CyclicPrompt (0.9454). Compared with earlier unified baselines such as All-in-One (31.12/0.9268) and WeatherDiff_64_ (30.71/0.9312), WeatherMAR still shows clear improvements in both metrics. These consistent improvements across diverse degradations support the effectiveness of our unified token-space formulation, in which paired-domain joint sequence modeling enables explicit cross-domain interaction and complementary bidirectional masking strengthens conditional learning within a single shared model.

#### 4.3.3. Qualitative Evaluation

**Figure 4:**
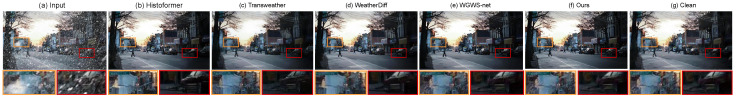
Visual comparison for image desnowing on Snow100K [8]. WeatherMAR achieves more complete snow removal across different snow severities and reconstructs sharper edges and finer textures, as highlighted in the selected regions. The red and orange boxes indicate representative local regions selected for detailed visual comparison. The zoomed-in patches shown below the main images correspond to these boxed regions, and the different box colors are used to distinguish the different selected regions.

**Figure 5:**
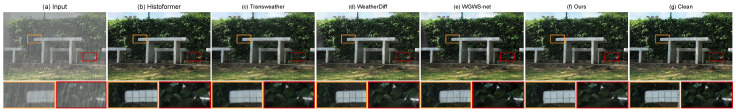
Visual comparison for joint deraining and dehazing on Outdoor-Rain [20]. WeatherMAR removes dense rain streaks and haze more thoroughly while better preserving scene structures, as highlighted in the zoomed regions. The red and orange boxes indicate representative local regions selected for detailed visual comparison. The zoomed-in patches shown below the main images correspond to these boxed regions, and the different box colors are used to distinguish the different selected regions. The red and orange boxes indicate representative local regions selected for detailed visual comparison. The zoomed-in patches shown below the main images correspond to these boxed regions, and the different box colors are used to distinguish the different selected regions.

**Figure 6:**
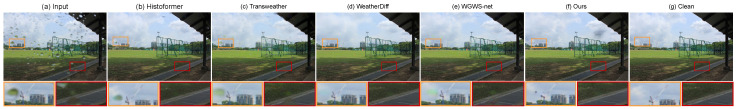
Visual comparison for raindrop removal on RainDrop [10]. Compared with prior methods, WeatherMAR suppresses adherent raindrops and restores the occluded background with fewer artifacts, as shown in the enlarged patches. The red and orange boxes indicate representative local regions selected for detailed visual comparison. The zoomed-in patches shown below the main images correspond to these boxed regions, and the different box colors are used to distinguish the different selected regions.

**Figure 7:**
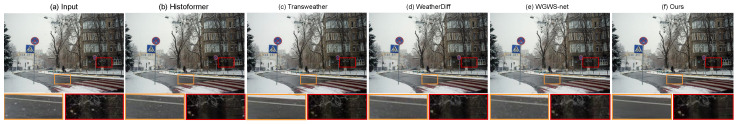
Qualitative results on the real-image subset Snow100K-Real [8]. Without ground truth, WeatherMAR produces cleaner snow-free images, preserves fine details, and avoids over-smoothing in challenging real-world scenes.

We further present visual comparisons on Snow100K, Outdoor-Rain, and RainDrop, together with real-world results on Snow100K-Real (Figure 4, Figure 5, Figure 6 and Figure 7). Overall, WeatherMAR produces cleaner restorations with fewer residual artifacts and stronger structural consistency, particularly in regions where degradations overlap with edges and fine textures. This behavior aligns with the unified token-space design, in which self-attention over the joint sequence enables cross-domain interaction and complementary masking promotes conditional completion rather than trivial recovery.

On **Snow100K**, as shown in Figure 4, WeatherMAR more thoroughly removes both sparse snow streaks and dense snow clusters in the zoomed regions. In areas with high-frequency textures (e.g., brick patterns, foliage, and repetitive structures), WeatherMAR preserves sharper boundaries and more coherent fine textures. Several competing methods leave thin snow residues, such as faint streaks and granular snow points, or suppress snow at the cost of over-smoothed surfaces. Around object contours and thin structures, WeatherMAR reduces snow remnants without blurring edges, yielding clearer outlines and fewer texture discontinuities in the highlighted patches.

On **Outdoor-Rain**, as illustrated in Figure 5, WeatherMAR reduces rain streaks and haze veiling, thereby improving visibility and global tonal consistency. In the enlarged crops, fine structures such as poles and wires are better preserved, with fewer halo artifacts around high-contrast transitions. Compared with baselines that under-remove rain or introduce local over-enhancement, WeatherMAR produces more balanced restorations by improving distant visibility while preserving local details.

On **RainDrop**, as shown in Figure 6, WeatherMAR suppresses raindrop boundaries and the associated refractive distortions. In raindrop-covered regions, WeatherMAR restores occluded content with fewer ringing artifacts near raindrop edges and fewer discontinuities in repeated textures. Competing methods often leave residual raindrop contours, produce locally inconsistent textures, or exhibit boundary ringing in magnified regions, whereas WeatherMAR yields more spatially coherent reconstructions.

On **Snow100K-Real**, WeatherMAR generalizes well to real snowy images despite the domain gap between synthetic training data and real-world snow patterns. As illustrated in Figure 7, WeatherMAR removes prominent snow deposits and streaks while preserving a natural appearance and avoiding common real-image desnowing failures such as over-smoothing, loss of fine textures, and color shifts. In challenging regions where snow partially occludes structural edges or overlaps textured backgrounds, WeatherMAR produces cleaner results with fewer residual traces and more coherent texture continuity.

In summary, Figure 4, Figure 5, Figure 6 and Figure 7 corroborate the quantitative trends in Table 1, showing that WeatherMAR suppresses diverse weather artifacts while preserving sharp edges and coherent textures within an adverse-weather restoration framework.

#### 4.3.4. No-Reference Quantitative Evaluation on Real Snow Images

To complement the qualitative results on Snow100K-Real, we additionally report no-reference image quality metrics to enable quantitative evaluation in the absence of paired ground truth. We compare against several representative unified baselines to provide a concise evaluation in the real-image setting. As shown in Table 2, WeatherMAR achieves the lowest NIQE and IL-NIQE scores among the compared methods, with values of 2.803 and 21.617, respectively. Compared with WeatherDiff_128_, WeatherMAR reduces NIQE from 2.964 to 2.803 and IL-NIQE from 21.976 to 21.617. These results provide additional evidence of the strong perceptual restoration quality of WeatherMAR on real snowy images.

### 4.4. Ablation Studies

#### 4.4.1. Component-Wise Ablation

To validate the contribution of each design component in WeatherMAR, we conduct component-wise ablations on Outdoor-Rain [20], where heavy rain and haze jointly challenge fine-detail recovery and structural consistency. Unless otherwise specified, all variants share the same KL-16 tokenizer [17], mar_large backbone [18], diffusion head, training budget, and inference iteration count *K*; only the specified component is modified.

We start from a conditional MAR baseline ($A_{0}$) performing iterative masked-token completion for clean tokens under $p_{\theta }\left(X\;|\;Y\right)$. Specifically, degraded tokens *Y* are provided as a fixed conditioning prefix, and the missing clean tokens indexed by $M_{x}$ are predicted through MAR parallel iterations. To avoid explicit paired-domain joint modeling, we adopt a unidirectional attention mask: clean tokens can attend to all degraded tokens and the visible clean tokens filled in during earlier iterations, whereas degraded tokens do not attend to clean tokens. This baseline masks only clean-domain tokens and excludes joint sequence modeling, complementary masking, reverse supervision, and ProS scheduling.

Table 3 reports the PSNR and SSIM results on Outdoor-Rain. Introducing paired-domain joint sequence modeling ($A_{1}$) yields consistent gains over $A_{0}$, indicating that self-attention over the unified sequence provides stronger cross-domain conditioning than external conditioning alone. Enabling complementary bidirectional masking ($A_{2}$) yields the largest improvement, increasing PSNR from 30.08 to 31.64 and SSIM from 0.9232 to 0.9367. This result supports the claim that the location-wise complementary constraint strengthens conditional completion by ensuring that, at each spatial position, one domain remains visible while the other must be inferred, thereby improving structural fidelity without increasing model capacity.

Adding reverse supervision by activating the auxiliary degradation-modeling loss on $M_{y}$ ($A_{3}$) further improves performance to 31.92/0.9396, suggesting that the reverse objective regularizes degradation-aware representations within the same backbone and token space. Finally, incorporating the progress-to-step schedule during inference ($A_{4}$) yields 31.91/0.9396. This component primarily improves efficiency by reallocating reverse diffusion steps across iterations according to inference progress (Equation (20)), while maintaining comparable restoration accuracy. Overall, these ablations indicate that WeatherMAR’s gains are driven primarily by complementary bidirectional masking and are further reinforced by joint sequence modeling, reverse supervision, and progress-to-step scheduling, yielding a unified restoration model that is both accurate and efficient on Outdoor-Rain.

#### 4.4.2. Masking Strategy Ablation

We further isolate the effect of the masking strategy using the same mar_large backbone under identical training and inference settings. Table 4 compares three masking variants: only-clean masking (standard conditional completion), independent masking of both domains, and the proposed complementary masking.

As shown in Table 4, independent masking ($B_{2}$) modestly improves over only-clean masking ($B_{1}$), as the model occasionally learns to predict missing tokens in both domains. However, i.i.d. masking permits two unfavorable cases at a given location: both tokens may be masked, which weakens local evidence and pushes prediction toward unconditional generation, or both tokens may remain visible, which encourages trivial solutions that bypass cross-domain reasoning. In contrast, complementary masking ($B_{3}$) eliminates both cases by enforcing $M_{y}=1-M_{x}$, thereby guaranteeing exactly one domain token is observed at each location. This location-wise constraint stabilizes paired-domain completion and yields the best restoration accuracy, substantially improving both PSNR and SSIM.

#### 4.4.3. Efficiency Analysis of ProS Scheduling

We further evaluate the proposed progress-to-step schedule (Section 3.5) as an inference-time efficiency optimization for WeatherMAR. Both variants use the same trained model, the same MAR inference procedure, and the same iteration count (K=64), differing only in how reverse diffusion steps are allocated. We report the total reverse-step budget, parameter count, peak inference memory, average per-image runtime, and restoration accuracy on Outdoor-Rain and Snow100K-L.

As shown in Table 5, ProS reduces the total reverse-step budget from 3200 to 1788 while keeping the model size unchanged. On both Outdoor-Rain and Snow100K-L, this yields a consistent runtime reduction of about 12% with nearly unchanged restoration accuracy. Since most competing restoration baselines do not report runtime under a unified implementation setting, we focus on a controlled within-model comparison. In this sense, ProS should be viewed as an efficiency optimization tailored to the MAR-style inference process of WeatherMAR. By reducing runtime to about 0.2 s per image, it also improves the practical usability of the model in real-world deployment.

#### 4.4.4. Token Prediction Head Ablation

We further compare two token prediction heads on Outdoor-Rain under the same WeatherMAR architecture and training protocol: a direct L2 regression head and the adopted diffusion head. This ablation examines whether continuous-valued token prediction in our framework is better modeled as deterministic regression or as conditional distribution modeling.

As shown in Table 6, replacing the diffusion head with direct L2 regression leads to a clear performance drop on Outdoor-Rain. Specifically, WeatherMAR improves PSNR from 31.24 to 31.91 and SSIM from 0.9315 to 0.9396. This result supports our choice of a diffusion-based token prediction head for continuous-valued visual tokens, indicating that deterministic regression is less effective than diffusion-based modeling for masked-token prediction in our framework.

#### 4.4.5. Key Hyperparameter Ablation

We further study three key hyperparameters of WeatherMAR: the complementary masking ratio *r*, the auxiliary loss weight λ, and the inference-time step range $\left(S_{\max},S_{\min}\right)$ used in the progress-to-step schedule. The results are summarized in Table 7.

For the training hyperparameters, the best performance is achieved at r=0.5, while both smaller and larger masking ratios lead to inferior results. This suggests that complementary masking is most effective when visible and masked information remain balanced across the paired domains. We use a unified masking ratio across all weather conditions, rather than tuning *r* separately for each degradation type. Although weather-specific tuning of *r* may further improve individual results, r=0.5 serves as a general and robust default setting for the unified multi-weather framework. For the auxiliary loss weight, performance consistently improves as λ increases from 0 to 1.0, indicating that reverse-direction supervision provides useful regularization and is most effective among the tested settings when assigned the same weight as the main objective.

For the inference-time step range, increasing the sampling budget yields only marginal gains in PSNR and SSIM while increasing runtime. In particular, (100,0) improves over (50,5) by only 0.04 dB in PSNR and 0.0003 in SSIM, while requiring longer inference time per image. By contrast, (25,5) is faster but causes a slight drop in accuracy. Therefore, we use $\left(S_{\max},S_{\min}\right)=\left(50,5\right)$ as the default setting, as it provides the best trade-off between restoration quality and inference efficiency.

#### 4.4.6. Higher-Resolution Feasibility Study

We conduct a supplementary feasibility study on higher-resolution inputs using Outdoor-Rain. Since WeatherMAR is trained under the standard 256 × 256 setting, full-image inference at larger resolutions would substantially increase the latent sequence length and the inference cost. We therefore adopt a simple overlapping patch-based inference strategy: each 720 × 480 image is processed using overlapping 256 × 256 patches with a nominal overlap of 64 pixels, and the restored patches are merged by averaging.

As shown in Table 8, extending WeatherMAR to 720 × 480 inputs leads to a moderate drop of 0.56 dB in PSNR and 0.0082 in SSIM relative to the standard 256 × 256 setting. Nevertheless, the framework remains effective under this higher-resolution setting, suggesting that WeatherMAR remains applicable to larger inputs under a simple patch-based inference strategy. This study serves as a feasibility check rather than a fully optimized high-resolution solution.

## 5. Discussion

### 5.1. Discussion on the Frozen KL-16 Tokenizer

In this work, we adopt a frozen KL-16 tokenizer as a shared and fixed image-to-token interface, following the standard continuous-token MAR [18] setup. This choice is also consistent with common practice in MAR/LDM-style latent generative frameworks [17], where KL-16 provides a practical balance between compression efficiency and reconstruction fidelity. Moreover, since WeatherMAR performs token prediction in a continuous latent space with a diffusion-based objective, the continuous representation produced by KL-VAE is naturally compatible with our modeling formulation.

Keeping the tokenizer frozen allows us to control this factor across all experiments, so that the observed performance gains can be attributed primarily to the proposed multi-weather restoration framework rather than to changes in tokenizer design or joint tokenizer optimization. For this reason, tokenizer choice is not treated as a primary research variable in the present study.

We note that different tokenizer settings, including compression ratios (e.g., KL-8 versus KL-16) and pretrained VAE weights, may influence the absolute restoration performance. However, such effects mainly reflect differences in latent representation quality, token sequence length, and modeling difficulty, and are shared by latent-token-based MAR-style methods in general rather than being specific to WeatherMAR. Therefore, the conclusions of this work should be understood as validating the effectiveness of WeatherMAR under a standard and controlled frozen KL-16 tokenizer setting, while broader tokenizer-level generalizability remains an important topic for future study.

More advanced tokenizers may further improve restoration quality, but a systematic study of tokenizer design lies beyond the scope of the present work and is better viewed as part of the broader development of continuous-token MAR. We leave this direction, together with stronger continuous-token MAR backbones, for future work.

### 5.2. Scope and Future Evaluation Directions

The scope of WeatherMAR should be understood within the standard multi-weather restoration benchmark setting, in which a common restoration framework is evaluated across representative adverse-weather benchmarks, namely Snow100K, Outdoor-Rain, and RainDrop, under their widely adopted training and evaluation protocols. Accordingly, the current experimental setting is consistent with established community practice in multi-weather image restoration.

At the same time, the present evaluation does not constitute exhaustive validation across all real-world adverse-weather conditions. Challenging cases such as mixed-weather scenes, unseen compound degradations, cross-dataset generalization, and low-light weather corruption remain beyond the main scope of this study. These settings are important for further assessing practical robustness and applicability, and we regard more realistic datasets and evaluation protocols covering mixed and unseen degradations as an important direction for future work.

### 5.3. Failure Cases and Limitations

**Figure 8:**
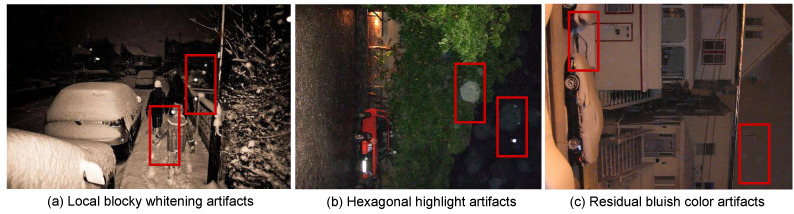
Failure cases of WeatherMAR on night-time real-snow scenes. The red boxes highlight representative failure regions, indicating the artifacts discussed in the caption, including local blocky whitening artifacts, hexagonal highlight artifacts, and residual bluish color artifacts in dark regions.

WeatherMAR may still fail in challenging night-time real-snow scenes. As shown in Figure 8, restoration errors may appear in dark regions, including incomplete snow removal, local blocky whitening, highlight-shaped residual patterns, and slight bluish color shifts. A likely reason is that low-light conditions provide weaker structural and textural cues in the degraded observations, thereby reducing the reliability of paired clean-token completion. In addition, real night-time snow scenes often involve headlight glare, sensor noise, reflections, and color distortion, making it more difficult to recover the underlying scene content from weather-corrupted observations. Improving robustness under low-light real-world snow conditions remains an important direction for future work.

## 6. Conclusions

We present WeatherMAR, an adverse-weather restoration framework that formulates restoration as paired-domain completion in a shared continuous token space. By jointly modeling degraded and clean tokens within a single sequence, WeatherMAR enables explicit cross-domain interaction through self-attention. Complementary bidirectional masking further strengthens location-wise conditional learning, while the progress-to-step schedule improves inference efficiency.

Experiments on Snow100K, Outdoor-Rain, and RainDrop demonstrate that WeatherMAR performs strongly across standard adverse-weather benchmarks, achieving the best results on Snow100K and RainDrop, as well as the best SSIM on Outdoor-Rain, among unified multi-weather restoration baselines. Additional analyses of real-image evaluation, efficiency, high-resolution feasibility, and failure cases further clarify the practical behavior and current scope of the framework. Overall, the results show that token-space completion with complementary masking provides an effective framework for multi-weather restoration under the standard benchmark setting. Extending the evaluation to more complex real-world degradations remains an important direction for future work. 

## Figures and Tables

**Table 1:** Quantitative comparison in terms of PSNR and SSIM (higher is better) on three adverse-weather restoration tasks: image desnowing (Snow100K-S/L), deraining and dehazing (Outdoor-Rain), and raindrop removal (RainDrop). The best and second-best results are shown in bold and underlined, respectively. The upper part reports task-specific methods, while the lower part presents unified multi-weather models, including WeatherMAR. Bold indicates the best result in each metric column. Underlined indicates the second-best result in each metric column.

Image Desnowing					Deraining & Dehazing			Raindrop Removal		
Method	Snow100K-S		Snow100K-L		Method	Outdoor-Rain		Method	RainDrop	
	PSNR	SSIM	PSNR	SSIM		PSNR	SSIM		PSNR	SSIM
SPANet [47]	29.92	0.8260	23.70	0.7930	CycleGAN [82]	17.62	0.6560	pix2pix [83]	28.02	0.8547
JSTASR [48]	31.40	0.9012	25.32	0.8076	pix2pix [83]	19.09	0.7100	DuRN [84]	31.24	0.9259
RESCAN [3]	31.51	0.9032	26.08	0.8108	HRGAN [20]	21.56	0.8550	RaindropAttn [11]	31.44	0.9263
DesnowNet [8]	32.33	0.9500	27.17	0.8983	PCNet [85]	26.19	0.9015	AttentiveGAN [10]	31.59	0.9170
DDMSNet [9]	34.34	0.9445	28.85	0.8772	MPRNet [77]	28.03	0.9192	IDT [76]	31.87	0.9313
NAFNet [86]	34.79	0.9497	30.06	0.9017	NAFNet [86]	29.59	0.9027	MAXIM [87]	31.87	0.9352
Restormer [88]	36.01	0.9579	30.36	0.9068	Restormer [88]	30.03	0.9215	Restormer [88]	32.18	0.9408
All-in-One [12]	–	–	28.33	0.8820	All-in-One [12]	24.71	0.8980	All-in-One [12]	31.12	0.9268
TransWeather [13]	32.51	0.9341	29.31	0.8879	TransWeather [13]	28.83	0.9000	TransWeather [13]	30.17	0.9157
Chen et al. [51]	34.42	0.9469	30.22	0.9071	Chen et al. [51]	29.27	0.9147	Chen et al. [51]	31.81	0.9309
WGWSNet [14]	34.31	0.9460	30.16	0.9007	WGWSNet [14]	29.32	0.9207	WGWSNet [14]	32.38	0.9378
WeatherDiff_64_ [15]	35.83	0.9566	30.09	0.9041	WeatherDiff_64_ [15]	29.64	0.9312	WeatherDiff_64_ [15]	30.71	0.9312
WeatherDiff_128_ [15]	35.02	0.9516	29.58	0.8941	WeatherDiff_128_ [15]	29.72	0.9216	WeatherDiff_128_ [15]	29.66	0.9225
AWRCP [16]	36.92	0.9652	31.92	**0.9341**	AWRCP [16]	31.39	0.9329	AWRCP [16]	31.93	0.9314
Histoformer [21]	37.41	0.9656	32.16	0.9261	Histoformer [21]	32.08	0.9389	Histoformer [21]	33.06	0.9441
T^3^-DiffWeather [89]	37.55	0.9641	31.11	0.9180	T^3^-DiffWeather [89]	32.52	0.9339	T^3^-DiffWeather [89]	32.70	0.9414
GridFormer [52]	37.46	0.9640	31.71	0.9231	GridFormer [52]	31.87	0.9335	GridFormer [52]	32.39	0.9362
CyclicPrompt [22]	37.50	0.9655	32.16	0.9265	CyclicPrompt [22]	**32.81**	0.9371	CyclicPrompt [22]	32.57	**0.9454**
WeatherMAR (Ours)	**38.14**	**0.9684**	**32.58**	0.9274	WeatherMAR (Ours)	31.91	**0.9396**	WeatherMAR (Ours)	**33.12**	0.9452

**Table 2:** No-reference image quality evaluation on Snow100K-Real using NIQE and IL-NIQE. Bold indicates the best result in the corresponding comparison. ↓ means lower is better.

Method	NIQE ↓	IL-NIQE ↓
TransWeather [13]	3.161	22.207
WeatherDiff_64_ [15]	2.985	22.121
WeatherDiff_128_ [15]	2.964	21.976
**WeatherMAR (Ours)**	**2.803**	**21.617**

**Table 3:** Component-wise ablation on Outdoor-Rain. Starting from a conditional MAR baseline ($A_{0}$), we progressively add paired-domain joint sequence modeling ($A_{1}$), complementary bidirectional masking with λ=0 ($A_{2}$), reverse supervision by enabling the auxiliary loss on $M_{y}$ with λ=1 ($A_{3}$), and progress-to-step scheduling for efficient inference ($A_{4}$). Bold indicates the best result in the corresponding comparison. ↑ means higher is better.

Method	PSNR ↑	SSIM ↑
$A_{0}$: Conditional MAR baseline (mar_large)	29.81	0.9204
$A_{1}$: + Joint sequence modeling (Equation (3))	30.08	0.9232
$A_{2}$: + Complementary masking (Section 3.3)	31.64	0.9367
$A_{3}$: + Reverse supervision (Equation (11))	**31.92**	**0.9396**
$A_{4}$: + Progress-to-step schedule (Equation (20))	31.91	**0.9396**

**Table 4:** Masking-strategy ablation on Outdoor-Rain. $B_{1}$ uses standard conditional completion by masking only clean tokens (the same setting as $A_{1}$ in Table 3). $B_{2}$ masks degraded and clean tokens independently (i.i.d.). $B_{3}$ applies complementary masking with $M_{y}=1-M_{x}$. Bold indicates the best result in the corresponding comparison. ↑ means higher is better.

Method	PSNR ↑	SSIM ↑
$B_{1}$: Only-clean masking (standard conditional; same as $A_{1}$)	30.08	0.9232
$B_{2}$: Independent masking (both domains, i.i.d.)	30.56	0.9288
$B_{3}$: Complementary masking ($M_{y}=1-M_{x}$)	**31.64**	**0.9367**

**Table 5:** Efficiency analysis of ProS scheduling on Outdoor-Rain and Snow100K-L. The fixed-step baseline uses $S_{k}=50$ at every iteration, whereas ProS uses the decreasing schedule in Equation (20). Both variants use the same WeatherMAR model and differ only in the allocation of reverse diffusion steps. ↓ means higher is better. ↑ means higher is better.

Method	Step Schedule	Total Steps	Params (M)	Mem (GB)	Time (s) ↓	Speed-Up ↑	PSNR/SSIM ↑
Outdoor-Rain							
WeatherMAR (fixed)	fixed $S_{k}=50$	3200	479	20.4	0.256	0.0%	31.92/0.9396
WeatherMAR + ProS	scheduled $S_{k}:50\;\to \;5$	1788	479	20.3	0.224	+12.5%	31.91/0.9396
Snow100K-L							
WeatherMAR (fixed)	fixed $S_{k}=50$	3200	479	22.5	0.287	0.0%	32.60/0.9274
WeatherMAR + ProS	scheduled $S_{k}:50\;\to \;5$	1788	479	22.3	0.250	+12.8%	32.58/0.9274

**Table 6:** Ablation of the token prediction head on Outdoor-Rain. Both variants share the same WeatherMAR architecture and differ only in the prediction head.

Variant	Token Prediction Head	PSNR/SSIM ↑
WeatherMAR-Reg	direct L2 regression	31.24/0.9315
WeatherMAR	diffusion head	31.91/0.9396

**Table 7:** Ablation of key hyperparameters on Outdoor-Rain. For *r* and λ, we report restoration accuracy under the same training and inference settings. For $\left(S_{\max},S_{\min}\right)$, we additionally report the average per-image inference time. ↓ means higher is better. ↑ means higher is better.

Parameter	Setting	Time (s) ↓	PSNR ↑	SSIM ↑
Masking ratio *r*	0.3	–	31.22	0.9335
	0.5	–	31.91	0.9396
	0.7	–	31.03	0.9312
Loss weight λ	0	–	31.64	0.9367
	0.5	–	31.85	0.9384
	1.0	–	31.91	0.9396
Step range $\left(S_{\max},S_{\min}\right)$	(25,5)	0.132	31.84	0.9387
	(50,5)	0.224	31.91	0.9396
	(100,0)	0.265	31.95	0.9399

**Table 8:** Higher-resolution feasibility study on Outdoor-Rain. WeatherMAR is additionally evaluated on 720 × 480 inputs using overlapping patch-based inference. ↓ means higher is better. ↑ means higher is better.

Method	Resolution	Inference Mode	PSNR ↑	SSIM ↑
WeatherMAR	256 × 256	standard inference	31.91	0.9396
WeatherMAR	720 × 480	patch-based inference	31.35	0.9314

## Data Availability

No new data were created or analyzed in this study.
